# Research on nurses’ prescription rights in China: A bibliometric analysis via CiteSpace

**DOI:** 10.1097/MD.0000000000049018

**Published:** 2026-05-29

**Authors:** Xiaoyu Zhang, Qiyuan Shi

**Affiliations:** a Nursing Teaching and Research Office, The First Affiliated Hospital of Heilongjiang University of Chinese Medicine, Harbin, Heilongjiang, China; b Third Treatment Section, Heilongjiang Compulsory Medical Treatment Institute, Harbin, Heilongjiang, China.

**Keywords:** bibliometric analysis, data visualization, nurses, prescription rights

## Abstract

**Background:**

At present, China has not established nationwide prescribing rights for nurses. Although pilot programs have been launched in a few regions and extensive discussions exist, no unified standard has been formed.

**Methods:**

Pertinent literature regarding nurses’ prescription rights was obtained from the China National Knowledge Infrastructure (CNKI) and Wanfang databases. Visual representations were generated through the analysis of publication year, author distribution, institutional affiliations, and keywords using CiteSpace 6.2R6 software.

**Results:**

A total of 348 publications were initially identified. Following thorough screening, 214 qualifying records were incorporated into the analysis. The yearly publication output demonstrated an increasing trend with variations, reaching a zenith in 2020 with 32 articles. The most prolific author was Han Shifan (n = 48), and the preeminent research institution was Shanxi Medical University (n = 62). Keyword analysis indicated the greatest frequency for terms including “nurses,” “prescription rights,” “nurse practitioners,” and “prescription forms” (n = 16). Keyword cluster analysis yielded 6 thematic categories: #0 Nurse, #1 Specialist Nurse, #2 Prescription Form, #3 Nurse Practitioners, #4 Nursing, and #5 Influencing Factors.

**Conclusions:**

Research on Chinese nurses’ prescription rights has evolved from initial explorations to a comprehensive understanding of its practical, legal, and educational implications. Early studies were mainly theoretical and limited in scope, while recent research features empirical analysis, detailed legal reviews, and structured educational frameworks.

## 1. Introduction

The prescription rights of nurses pertain to their authority to identify the most suitable therapeutic interventions for particular health conditions.^[1]^In 1970, the American Nurses Association (ANA) officially defined the responsibilities of nurses regarding medication prescription.^[2]^Since that time, the extent of nurses’ prescribing authority has broadened internationally.^[3]^As of 2019, nurses in 40 countries and territories possessed prescription rights,^[4]^ a number that rose to 44 by 2021.^[5]^In countries like Australia, the United States, and the United Kingdom, nurse practitioners and midwives have obtained prescription rights, enabling comprehensive examination of numerous facets of this practice, including legislation, scope, content, eligibility criteria, and training.^[6,7]^

Conversely, nurses in China presently do not possess prescription rights according to the current Nurses’ Regulation. This limitation is mainly due to apprehensions regarding patient safety, the necessity for standardized medical protocols, and the legal difficulties or professional responsibility of the healthcare system. The changing healthcare environment and the rising demand for effective medical services have initiated discussions regarding the authorization of prescription rights for nurses.^[8]^ Anhui Province commenced a pilot program for nurse prescription rights in 2017,^[9]^ while Shenzhen City has furthered this initiative by conferring limited prescription authority to specialist nurses in traditional Chinese medicine (TCM).^[10]^

Extending prescription authority to nurses could improve patient outcomes, stabilize the nursing workforce, and highlight the professional significance of nursing. Research indicates that nurse-prescribed treatments are as effective as those prescribed by physicians, with patient satisfaction frequently equal to or exceeding that of physician care.^[11,12]^ Consequently, academic interest in the prescription rights of nurses has increased in China. Nevertheless, limited research has utilized literature visualization methods to thoroughly investigate this subject.^[13]^ In the context of studies on nurses’ prescription rights in China, the majority of research efforts have been concentrated on traditional qualitative and quantitative analyses, such as narrative reviews, statistical hypothesis testing, and meta - analyses. While these methods have provided valuable insights into the current state and trends, they often fall short in presenting the complex relationships and knowledge networks within the research field in a more intuitive and comprehensive manner.

This study collected research articles regarding nurses’ prescription rights from the China National Knowledge Infrastructure (CNKI) and Wanfang databases. The CiteSpace visualization software was employed to analyze complex data via graphical and tabular representations, thereby identifying research hotspots and emerging trends in the field. The results seek to establish a basis for subsequent research and guide the formulation of pertinent policies.

## 2. Data and methods

This study utilized bibliometric analysis of existing literature and did not conduct direct experimentation on humans or animals. Therefore, ethical approval was not required.

### 2.1. Data sources

In January 2024, a comprehensive search was performed in the China National Knowledge Infrastructure (CNKI) database utilizing the search formula SU = “nurses” AND “prescription rights” with the stipulation of “exact.” This search obtained all journal articles published from the database inception to December 2023, resulting in 229 articles. An analogous advanced search in the Wanfang database employing the formula SU = “nurses” AND KY = “prescription rights” with the “fuzzy” search criterion yielded 119 articles. The complete dataset consisted of 348 articles.

### 2.2. Data processing methods

#### 2.2.1. Literature processing workflow

**Figure 1.**
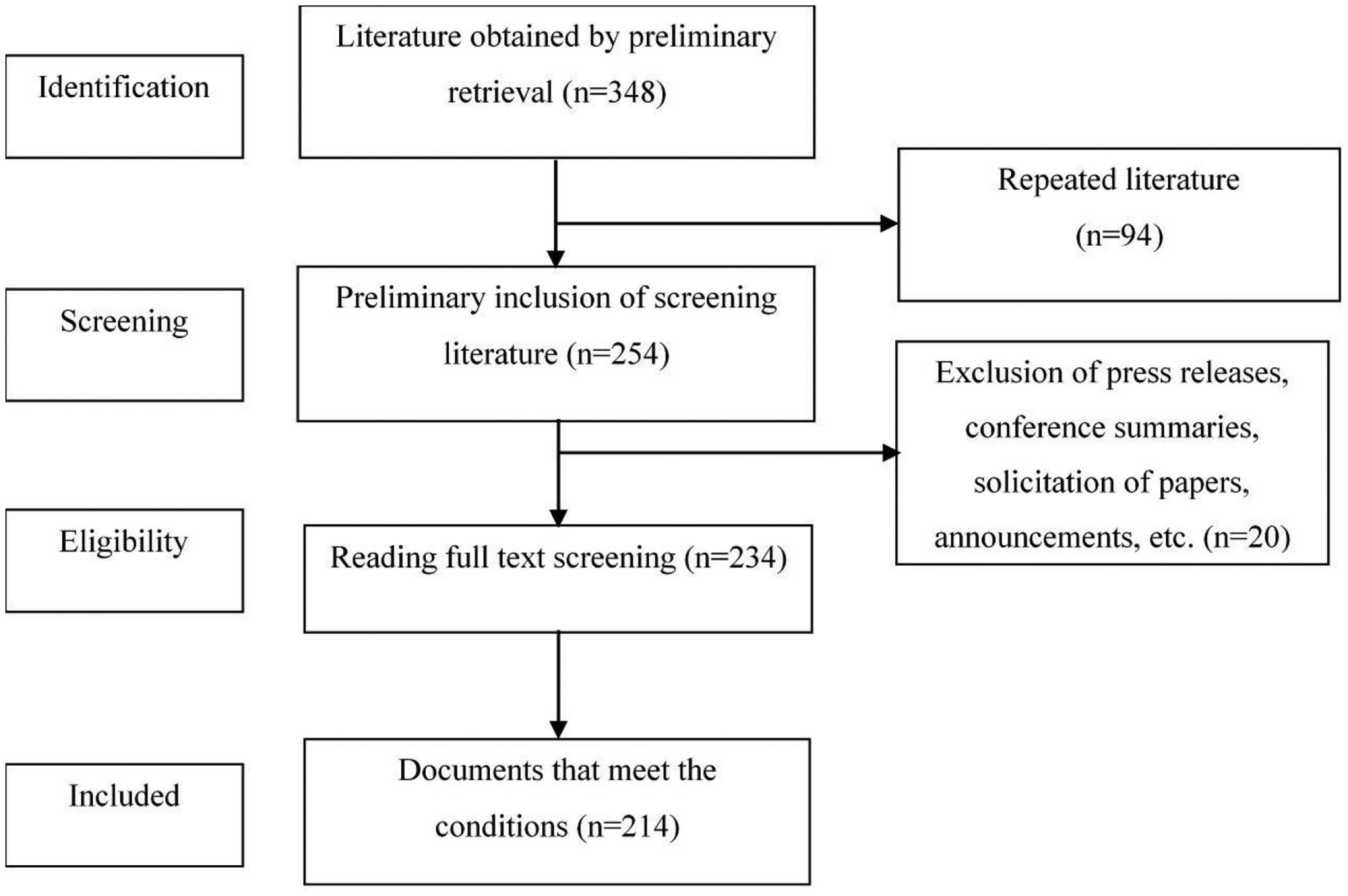
The Literature selection process. Literature selection process for nurse prescriptive authority research (2000–2023).

The search results were exported in RefWorks format and subsequently imported into Note Express v4.0.0 software for enhancement. One hundred twenty-four records were excluded due to criteria including duplication, absence of authorship, irrelevance to the research topic, or classification as conference abstracts, news reports, or other non-qualifying formats. Following the screening process, 214 articles were selected for analysis. Figure 1 illustrates the comprehensive workflow for literature processing.

#### 2.2.2. Data analysis procedures

The curated dataset was modified and formatted utilizing Note Express v4.0.0 software, and records were exported in the RefWorks-CiteSpace format. The analysis encompassed the timeframe from January 2000 to December 2023, with temporal segmentation established at 1 year. The chosen node types comprised author, institution, and keyword. Essential study attributes—including publication frequency, author dispersion, institutional connections, keywords, and emerging terminology—were examined to create a visual co-occurrence knowledge graph.

#### 2.2.3. The disadvantages and limitations of CiteSpace

CiteSpace analysis heavily relies on the input data. If the data comes solely from specific databases such as Web of Science or China National Knowledge Infrastructure, it may be limited by the database coverage, leading to the omission of important documents from other data sources, thus resulting in biased analysis. The quality of raw data directly affects the reliability of CiteSpace analysis results. Issues such as data entry errors, nonstandard labeling, or missing key information can mislead the analysis.

## 3. Results

### 3.1. Annual bibliometric analysis

**Figure 2.**
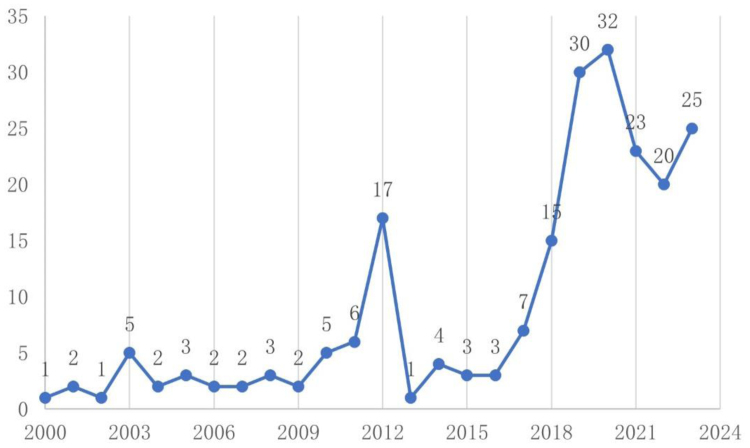
Number of articles issued, FY2000–FY2023. Statistical analysis of the number of published articles in the field of nurse prescriptive authority (FY2000–FY2023).

Figure 2 illustrates the annual distribution of publications. Investigations into the prescription rights of nurses in China indicate a general upward trajectory. Before 2011, research in this domain was constrained, probably owing to insufficient awareness and resources. A slight rise in 2012, succeeded by a downturn, may indicate the conclusion of short-term research initiatives. Subsequent to 2017, there was a consistent increase in publications, signifying an escalating scholarly interest, likely influenced by continuous healthcare reform initiatives and the professional advancement of the nursing workforce.

### 3.2. Bibliometric analysis of authors

**Figure 3.**
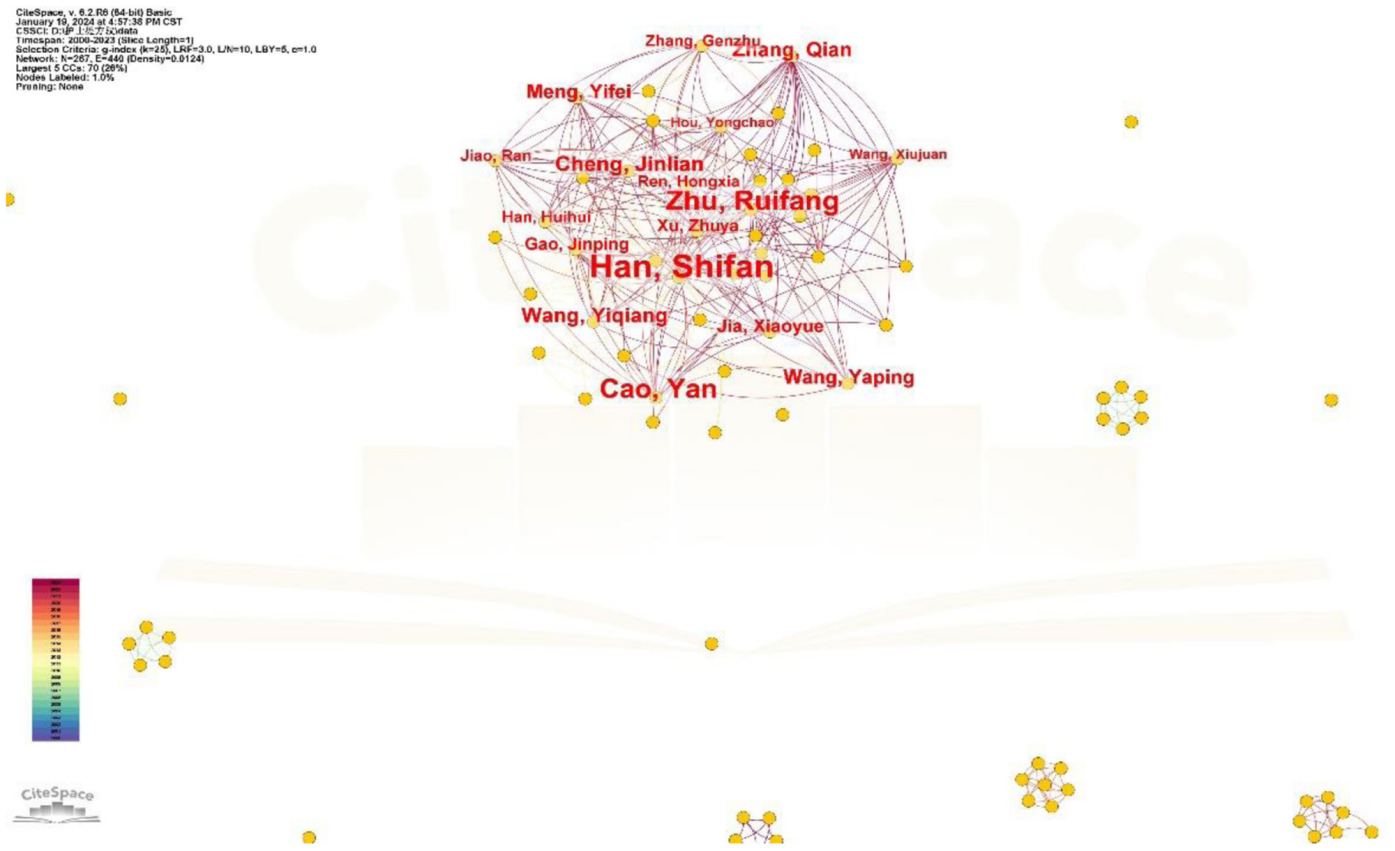
Author collaboration network map in China. Author collaboration network map of nurse prescriptive authority research in China.

A bibliometric network analysis conducted with CiteSpace 6.2.R6 identified a network comprising 267 nodes and 440 edges (density = 0.0124). Prominent contributors, such as Han Shifan (48 publications), Zhu Ruifang (25 publications), and Cao Yan (22 publications), have significantly advanced this field. Han Shifan has conducted extensive research, encompassing international comparisons and domestic strategies for the implementation of nurses’ prescription rights. Table 1 shows the author posts. Figure 3 illustrates the collaborative connections among the primary authors.

**Table 1:** Author posts.

Authors	Year	Centrality	次数
Han, Shifan	2010	0.03	48
Zhu, Ruifang	2017	0	25
Cao, Yan	2018	0	22
Cheng, Jinlian	2010	0	11
Wang, Yiqiang	2010	0	10
Zhang, Qian	2021	0	9
Wang, Yaping	2020	0	8
Meng, Yifei	2019	0	8
Jia, Xiaoyue	2020	0	7
Gao, Jinping	2020	0	6
Xu, Zhuya	2020	0	6
Ren, Hongxia	2019	0	5
Zhang, Genzhu	2012	0	5
Jiao, Ran	2019	0	5
Han, Huihui	2010	0	5

Core authors and their publication output in nurse prescriptive authority research.

### 3.3. Bibliometric analysis of institutions

**Figure 4.**
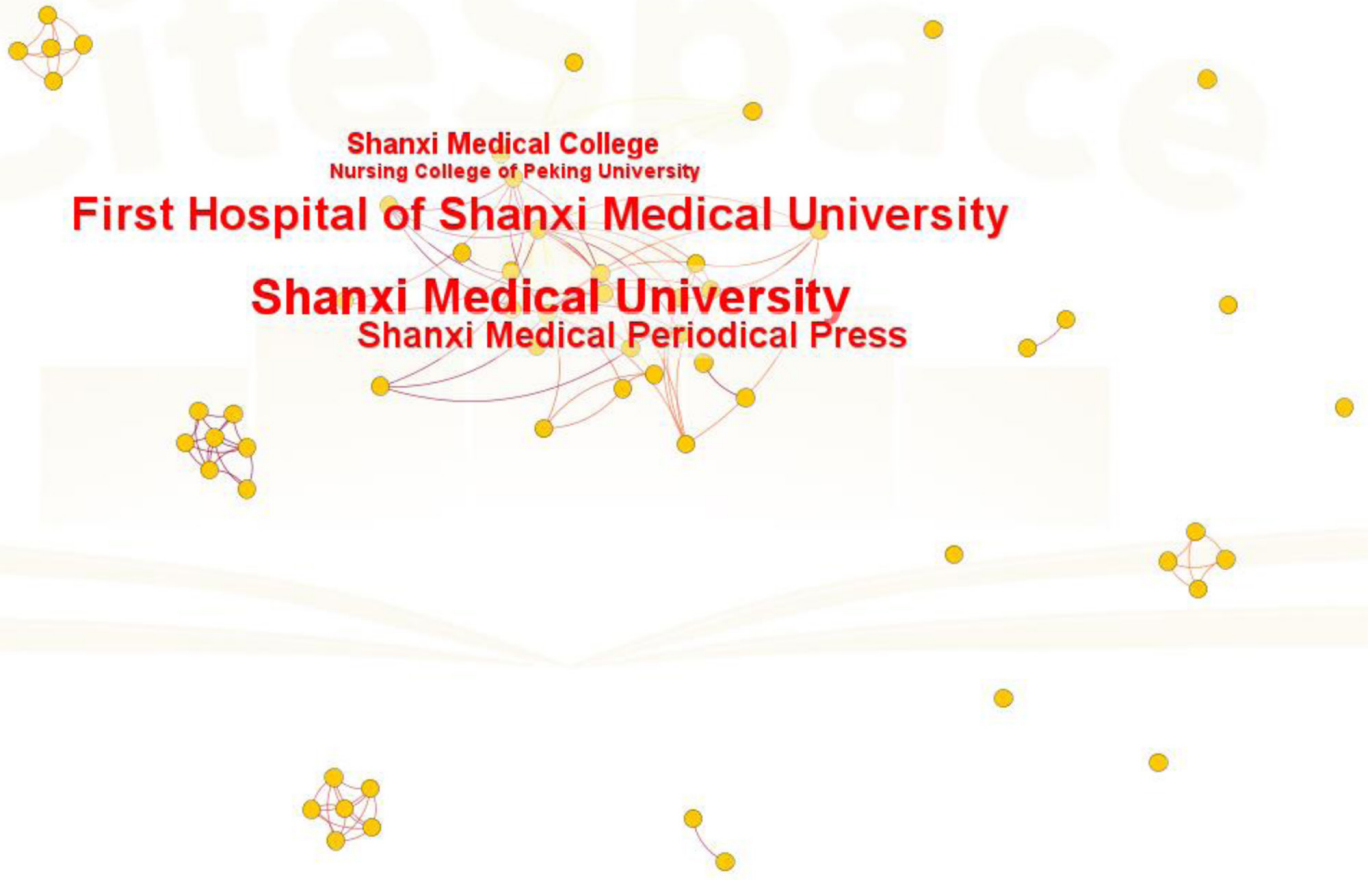
Institutional collaboration network map in China. Institutional collaboration network map of nurse prescriptive authority research in China.

The institutional collaboration network consisted of 195 nodes and 169 edges (density = 0.0089). Shanxi Medical University (62 publications) and the First Hospital of Shanxi Medical University (47 publications) have established themselves as prominent research institutions. These institutions have made significant initial contributions, fostering collaboration and generating impactful studies. Other institutions, albeit more dispersed, enhance the diversity of perspectives within this domain.Table 2 shows the authors’ affiliations. Figure 4 depicts the institutional collaboration network.

**Table 2:** Research institutions with higher volume.

Name of organization	Year	Centrality	N
Shanxi Medical University	2010	0.02	62
First Hospital of Shanxi Medical University	2010	0.01	47
Shanxi Medical Periodical Press	2018	0	20
Shanxi Medical College	2010	0	9
Nursing College of Peking University	2010	0.01	4
School of Nursing at Peking Union Medical College	2008	0	3

VolumeDistribution of high-output research institutions in the field of nurse prescriptive authority.

### 3.4. Bibliometric analysis of journals

A total of 179 journals were examined, with 2 journals publishing over 30 pertinent articles: Chinese Nursing Research (36 articles) and Chinese General Practice Nursing (30 articles). Table 3 delineates the distribution of articles among journals, emphasizing the principal publications that contribute to this research domain.

**Table 3:** Distribution of the published journals.

Journal Name	volume of periodical (n)
Chinese Nursing Research	36
Chinese General Practice Nursing	30
Chinese Journal of Nursing	8
Journal of Nursing Science	6
China Health Human Resources	6
Chinese Nursing Management	6
Journal of Nurses Training	6
Nursing Foreign Medical Sciences	5
Chinese Journal of Nursing Education	4

Journal distribution of published literature related to nurse prescriptive authority.

### 3.5. Analysis of co-occurrence keyword

**Figure 5.**
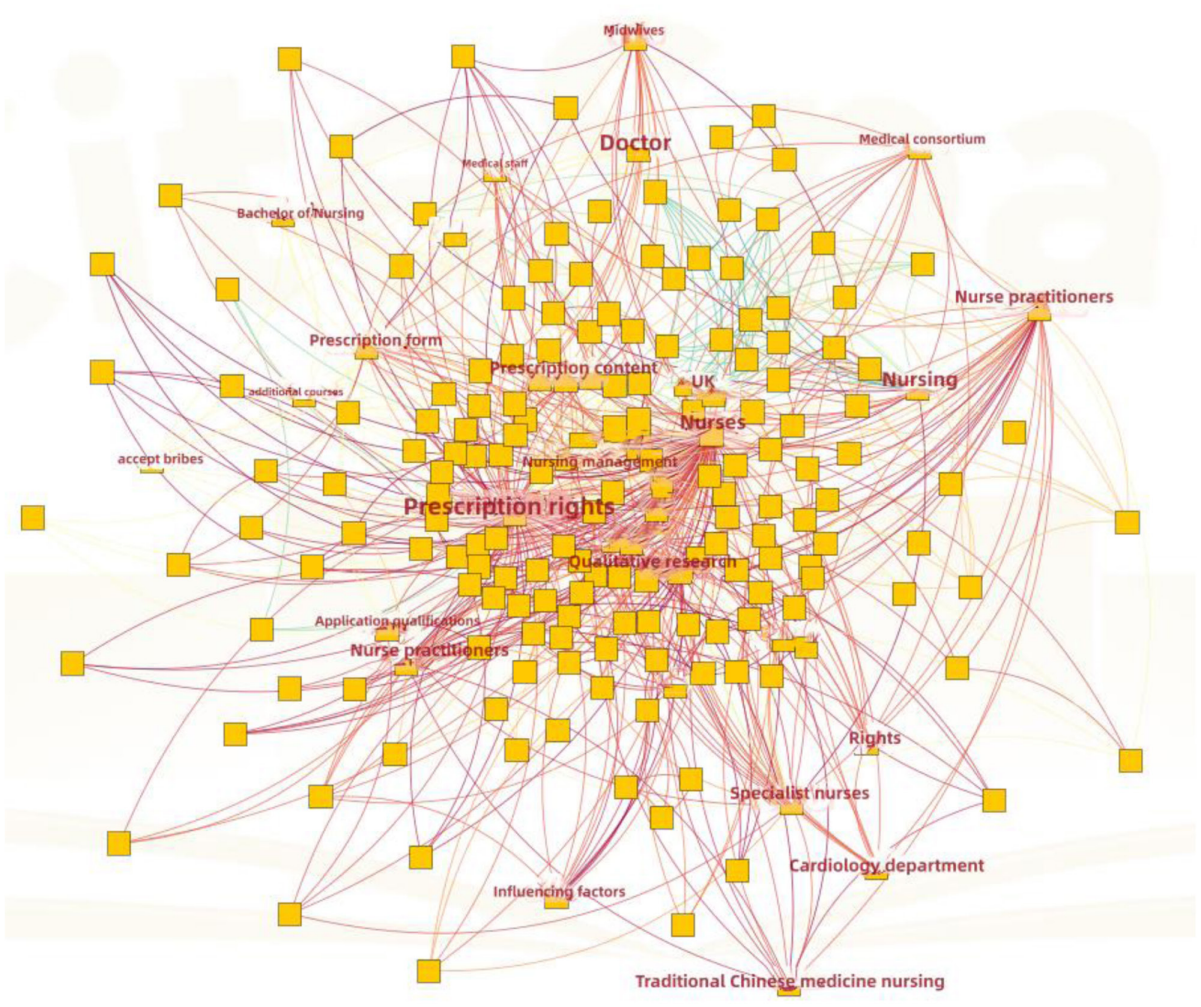
The keyword co-occurrence map from 2000 to 2023 in China. Keyword co-occurrence map of nurse prescriptive authority research in China (2000–2023).

CiteSpace 6.2.R6 was employed to consolidate synonymous keywords, thereby reducing redundancy and emphasizing essential concepts. For example, the terms “nurse,” “nursing staff,” “nurse practitioner,” and “clinical nurse” were amalgamated, as were “prescription content” and “content of prescription authority.” Keywords devoid of significant meaning, such as “review” and “research progress,” were omitted. The network comprised 226 nodes, 713 edges, and exhibited a density of 0.028 (Fig. 5). The analysis of keyword frequency produced 585 entries, with the top 10 keywords presented in Table 4.

**Table 4:** High frequency keywords.

Keyword	Frequency	Centrality	Year of occurrence
Prescription rights	97	0.68	2005
Nurse	83	0.49	2005
Opening nurse practitioners	17	0.16	2010
Prescription form	16	0.01	2011
Specialist Nurse	16	0.12	2009
Nursing clinic	15	0.07	2012
nursing	14	0.16	2003
Midwife	12	0.03	2017
Qualitative research	9	0.08	2017
Specific situation	9	0	2011

Statistics of high frequency keywords in nurse prescriptive authority research.

**Figure 6.**
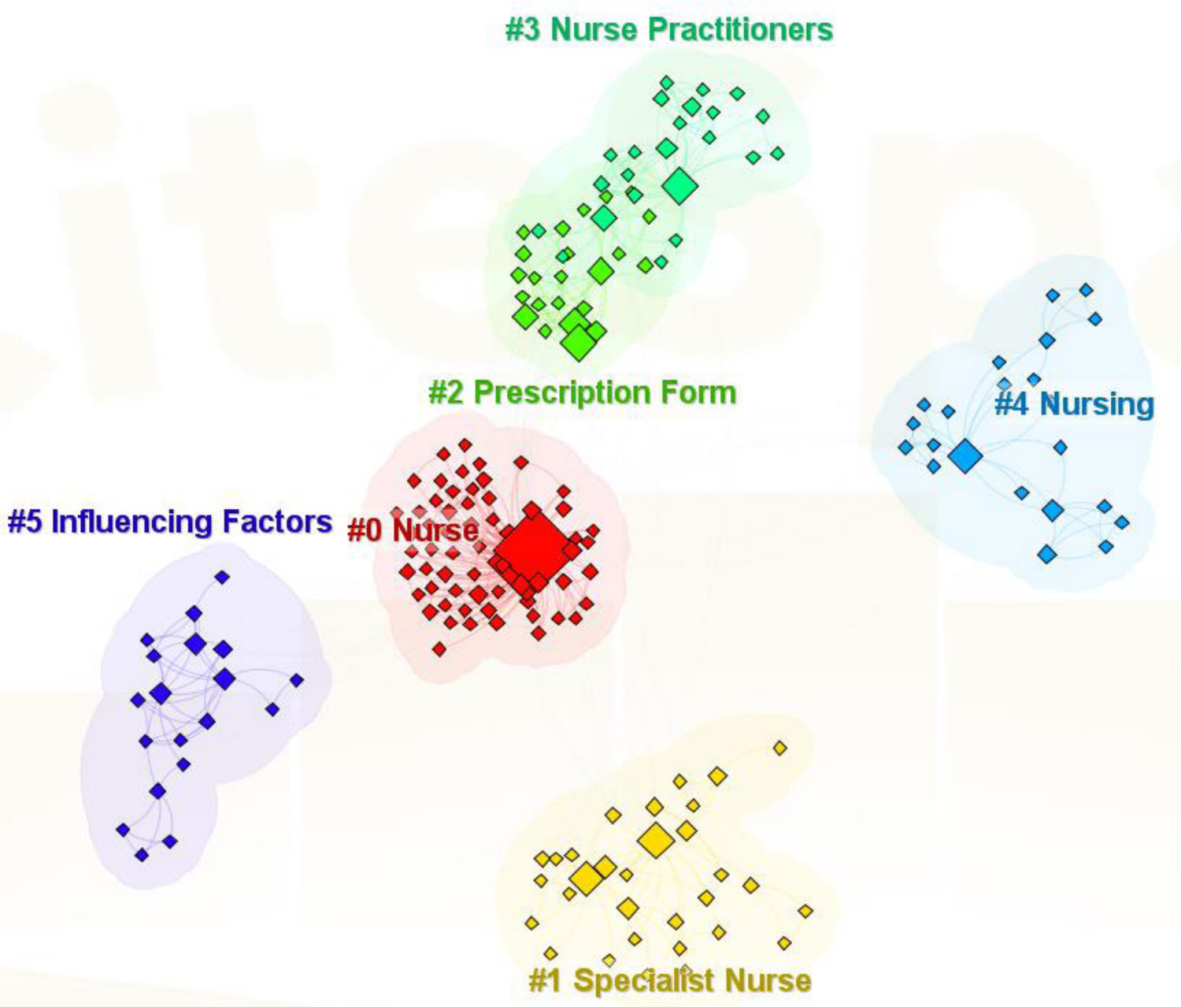
Cluster analysis of nurses’ prescriptive authority study. Cluster analysis results of nurse prescriptive authority research themes.

A clustered keyword knowledge graph (Fig. 6) delineated 6 primary clusters: #0 Nurse, #1 Specialist Nurse, #2 Prescription Form, #3 Nurse Practitioners, #4 Nursing, #5 Influencing Factors. Clusters with lower numerical labels encompassed a greater number of keywords, signifying their significance in the dataset.

### 3.6. Research trends

**Figure 7.**
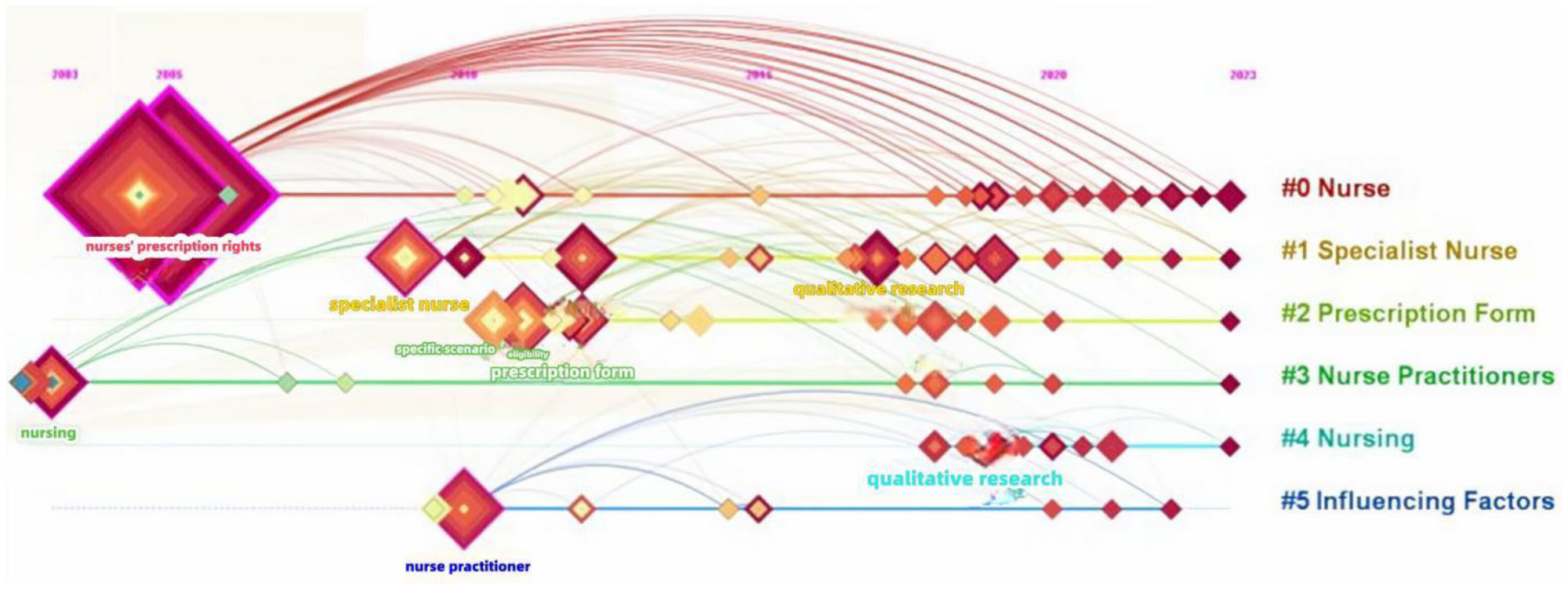
Timeline distribution of nurse prescriptive authority studies. Timeline distribution of nurse prescriptive authority research (2000–2023).

**Figure 8.**
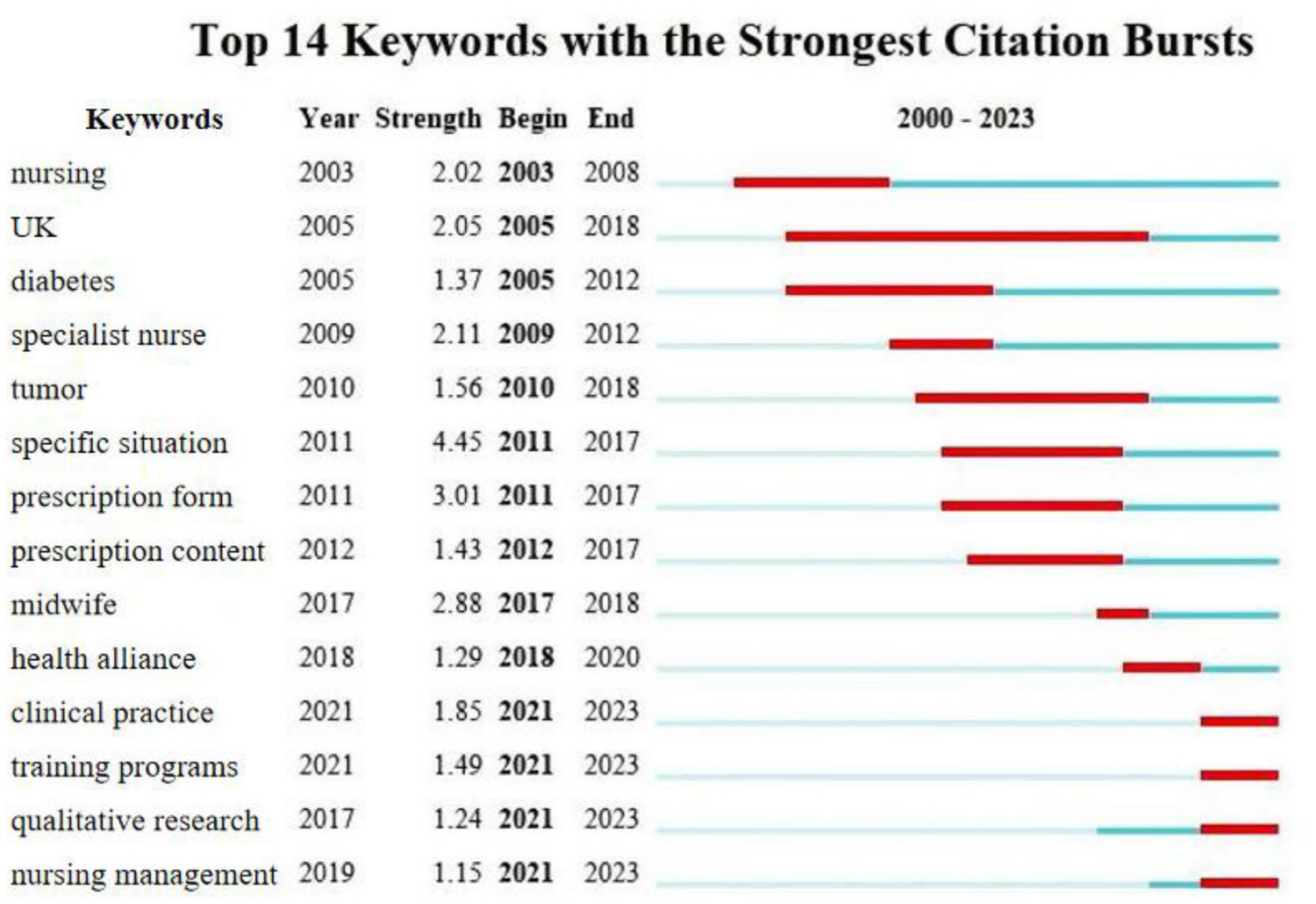
Top 14 keywords with the strongest citation bursts in China between 2000–2023. Top 14 keywords with the strongest citation bursts in Chinese nurse prescriptive authority research (2000–2023).

The timeline distribution of keyword clusters demonstrates the evolution of research themes (Fig. 7), whereas Figure 8 emphasizes the dynamic interaction of topics concerning nurses’ prescription rights.

Initial research predominantly concentrated on assimilating international practices (e.g., from the UK, US, and Australia) and examined particular nursing domains in China, including diabetes, oncology, and cardiology care. Subsequently, research broadened to investigate the allocation of prescription authority in particular contexts, the formats and substance of nurse-generated prescriptions, and legal implications.

Since 2014, research has progressively focused on the legal framework and execution of prescription rights for nurses. Current research focal points have concentrated on pragmatic applications, including clinical practice, training program design, qualitative research methodologies, and innovations in nursing management.

## 4. Discussion

Compared with the previous research on the prescription right of Chinese nurses, which was mainly based on theoretical discussion and limited pilot projects, this survey represents a substantial leap. The early research mainly discussed the theoretical feasibility of the prescription right of nurses, and some even explored the pilot projects in specific regions. This paper presents and summarizes the previous research content more intuitively.

### 4.1. Trends in communication

The notion of nurses’ prescription rights was initially presented in national journals in 1986.^[14]^ Nevertheless, research on this subject was scarce until 2011, averaging merely 2.83 publications per year.^[15]^ The minimal scholarly activity continued despite China significant population of over 2 million registered nurses by the end of 2011, indicating a widespread deficiency in academic involvement with this issue in earlier years.

In 2012, research output significantly increased, totaling 17 publications, which included 5 dissertations and 12 journal articles. Notable contributors during this period comprised Han Shifan (9 articles), Cheng Jinlian (5 articles), and Wang Yiman (5 articles). Commonly referenced terms, including “prescription rights” (7 instances), “Delphi method” (6 instances), and “specialized nurses” (5 instances), indicate the scholarly emphasis of this period. The research examined multiple aspects of nurse prescribing, encompassing qualification requirements, prescription specifics for distinct nursing roles (e.g., emergency, diabetes, and oncology care), and comparative evaluations of international practices in countries like the United States and the United Kingdom.

By 2013, publication activity experienced a significant decline, resulting in the production of only 1 article. This decline can be ascribed to multiple factors, such as the conclusion of prior research initiatives, inadequate funding for new investigations, and the lack of specialized platforms for scholarly exchange in this nascent discipline. Such constraints impeded the continuous progress of research.^[16]^

Since 2017, a consistent rise in academic output has been noted, indicating an increasing Acknowledgments of the changing functions of nurses and the necessity to enhance healthcare resources. Research conducted during this period has encompassed notable contributions, including SWOT analyses of nurse prescribing in China,^[17]^ regional studies (e.g., Anhui Province), and deliberations on legislative frameworks governing nurse prescription rights.^[18]^ Researchers including Qian Lijuan,^[19]^ Xing Mengting,^[20]^ and Wang Long^[21]^have achieved significant progress by analyzing the practical, systemic, and legislative aspects of conferring prescription rights to nurses.This result is similar to previous studies.^[13]^

### 4.2. Analysis of major research institutions and core authors

The foremost research institutions in this domain are situated in Shanxi Province, with Shanxi Medical University serving as a pivotal center. This preeminence is evident in the works of prolific authors like Han Shifan, Zhu Ruifang, and Cao Yan, all associated with Shanxi Medical University.

Han Shifan and his team have made substantial progress in this field through extensive research that incorporates both international and domestic viewpoints. Their research has examined essential topics, including the justification for granting nurses prescription authority, the related advantages and challenges, qualification standards, and prescription specifics in diverse clinical contexts, such as emergency care and chronic disease management. In addition to clinical practice, the team has significantly contributed to education by promoting the incorporation of nurse prescribing courses in both undergraduate and graduate nursing programs. This integrative approach demonstrates a dedication to improving nurses’ professional competencies while tackling wider public health requirements.

### 4.3. Hotspots and research frontiers in nurse prescriptive authority

Keyword analysis offers a succinct overview of principal themes and emerging trends in the examination of nurses’ prescription rights.The results were significantly different from previous studies, which may be due to different inclusion of literature and selection time.^[13]^. Results from CiteSpace 6.2.R6 indicate that research has become increasingly deepened over time.

Research Focus: Investigations have analyzed certification frameworks for specialized practice nurses, including midwives, oncology nurses, and gynecology and obstetrics nurses.^[22,23]^ Nurses in certain fields have restricted prescription authority, yet the relevant legal frameworks are inadequately developed.^[24,25]^

Content Development: Research has evolved from summarizing global experiences to focusing on the particular needs of China, including the management of acute conditions (e.g., anaphylactic shock and cardiac arrest) and chronic diseases (e.g., diabetes and hypertension).^[26]^ Current discussions encompass debates regarding independent vs protocol-driven prescriptions and the specifics of nurse-issued prescriptions. Standardized training programs for nurse prescribers have also been developed in China.^[27]^

Methodological Evolution: Initial research primarily relied on literature reviews and research methodologies such as the Delphi method. Recent research has adopted a variety of approaches, including qualitative studies centered on nursing outpatient services,^[28]^evidence-based clinical practice systems for nonconventional methods,^[29]^ and specialist nurses in TCM.^[30]^

Emerging research frontiers, depicted in Figure 8, encompass themes such as “clinical practice,” “training programs,” “qualitative research,” and “nursing management.” These advancements are propelled by China Healthy China 2030 initiative, the challenges posed by an aging demographic, and the increasing incidence of chronic illnesses. Extending prescription privileges to nurses is increasingly regarded as a method to mitigate healthcare resource inequities, improve patient outcomes, elevate nurse job satisfaction, and decrease workforce turnover.

## 5. Limitations

A significant limitation of this study is the lack of a collaborative network map among countries, which illustrates the distinct attributes of China healthcare system and its deviation from global frameworks. Presently, investigations into nurse prescription rights exhibit a deficiency in international collaboration owing to considerable disparities in legal and medical frameworks.

## 6. Conclusion

This bibliometric analysis elucidates the present status and trends in research regarding nurses’ prescription rights in China. Current research on nurses’ prescription rights in China focuses on “clinical practice,” “training programs,” “qualitative research,” and “nursing management,” laying a foundation for future studies and policy - making. This bibliometric analysis identifies existing knowledge, points out uncertain areas, and suggests research directions, thus advancing evidence - based policies and practices for nurses’ prescription rights.

## Acknowledgments

We would like to express our gratitude to Chao-Mei Chen from Drexel University in the United States for the development of the CiteSpace software and its distribution for free online.

## Author contributions

**Conceptualization:** Xiaoyu Zhang.

**Formal analysis:** Xiaoyu Zhang, Qiyuan Shi.

**Methodology:** Xiaoyu Zhang.

**Project administration:** Xiaoyu Zhang.

**Visualization:** Xiaoyu Zhang, Qiyuan Shi.

**Writing—original draft:** Xiaoyu Zhang.

**Writing—review & editing:** Xiaoyu Zhang, Qiyuan Shi.
